# A Case of Acute Prosthesis Migration after Femoral Head Replacement due to Osteomalacia by FGF23-Induced Tumor

**DOI:** 10.1155/2012/503956

**Published:** 2012-11-27

**Authors:** Shinya Hayashi, Takayuki Nishiyama, Takaaki Fujishiro, Shingo Hashimoto, Noriyuki Kanzaki, Teruya Kawamoto, Toshihiro Akisue, Kotaro Nishida, Masahiro Kurosaka

**Affiliations:** Department of Orthopaedic Surgery, Graduate School of Medicine, Kobe University, 7-5-1 Kusunoki-cho, Chuo-ku, Kobe 650-0017, Japan

## Abstract

Fibroblast growth factor 23 (FGF23) was recently identified as an important factor involved in the development of hypophosphatemic rickets and osteomalacia. We experienced a rare case of acute prosthesis migration after hemihip arthroplasty due to FGF23-induced tumor. The patient underwent femoral head replacement because of femoral neck fracture, but prosthesis migration was occurred at 1 week after operation. The patient took various examinations, and FGF23-induced tumor was found in his right wrist. The tumor was resected, and he underwent total hip arthroplasty 8 month later. Finally, he was able to obtain free gait without pain.

## 1. Introduction

FGF23 was recently identified as an important factor involved in the development of hypophosphatemic rickets and osteomalacia [1, 2]. It is associated with a phosphaturic mesenchymal tumor of mixed connective tissue located in the bone or soft tissue. The biochemical features include renal phosphate loss, low serum phosphate and 1,25-(OH)2 vitD3 levels, increased alkaline phosphatase, and normal calcium, PTH, calcitonin, 25-OH-vitD3, and 25,25-(OH)2 vitD3 [3]. Acute femoral prosthesis migration due to osteomalacia has never been reported. Here, we presented a case of acute prosthesis migration after hemihip arthroplasty due to osteomalacia by FGF23-induced tumor.

## 2. Case Presentation

**Figure 1:**
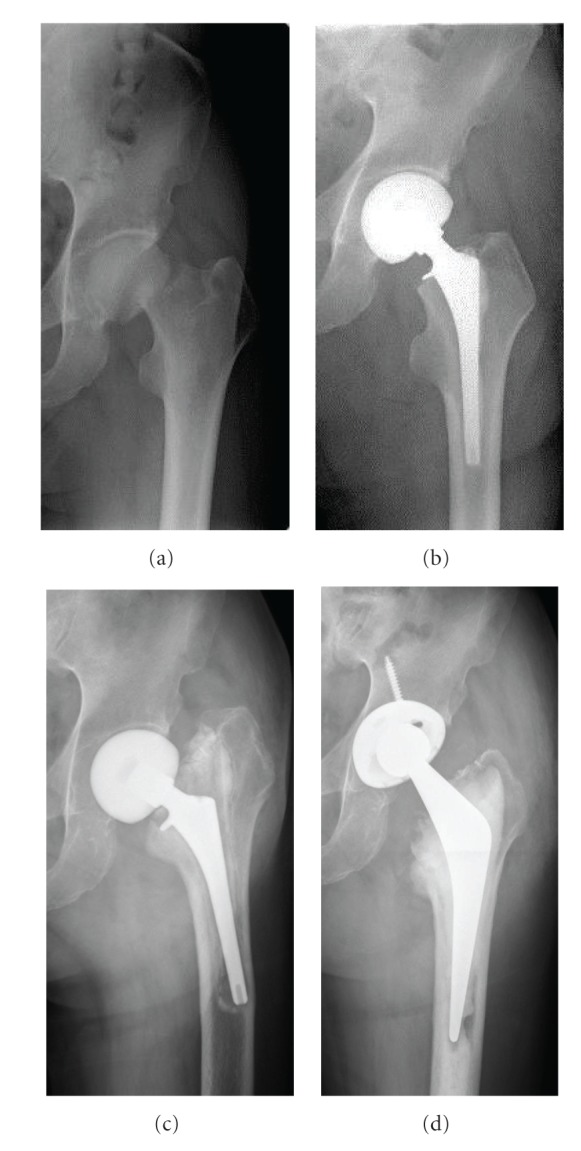
Radiographical findings, (a) before operation of femoral head replacement, (b) after operation of femoral head replacement, (c) 1 week after operation of femoral head replacement.

**Figure 2:**
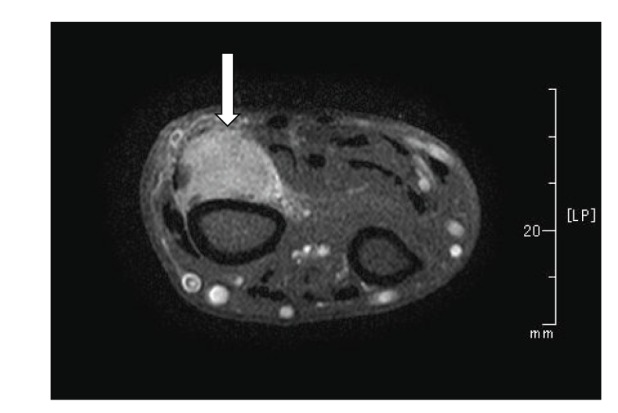
Gadolinium enhanced MRI image of the patient's right wrist.White arrow indicates the tumor.

A 66-year-old man was fallen down from ladder and was unable to stand up. He was taken emergency transportation to high care unit. He did not have any signs other than his left hip preoperatively. X-ray finding revealed the left femoral neck fracture, and the bone density of proximal femur was normal (Figure 1(a)). The operation of femoral head replacement was performed 2 days later. Intraoperative findings showed that the quality of bone was not so fragile during cutting femoral neck and stem insertion. The surgeons used cemented stem with third-generation cementing technic, but did not try press fit during component trial and stem insertion. Therefore, they did not verify the actual bone quality. They confirmed that there was no evidence of intraoperative fracture within the remnant of the femoral neck. X-ray revealed that the alignment of stem was normally straight. Actually, after operation X-ray showed that the cement mantle was not enough. However, that stability of stem was good after implantation (Figure 1(b)). They did not consider that the fracture was caused by osteomalacia due to tumor. Therefore, they did not send femoral head to pathology. Walking with full weight bearing was allowed immediately. However, he began to feel thigh pain during walking. The X-ray at one week later after operation showed the alignment of stem had been varus (Figure 1(c)). That finding means stem migration. There was no family or personal history of bone disease. Blood examination at the time of admission showed low serum phosphate (1.3 mg/dL) and increased alkaline phosphatase (650 U/L). He had been consultation to endoclinology in our hospital. The patient was taken bone mineral density (BMD) and blood examination of calcium, serum 25-(OH)2 vitD3, PTH, calcitonin, 25-OH-vitD3 and 25,25-(OH)2 vitD3. The value of BMD was normal. The concentration of serum 25-(OH)2 vitD3 was low, and calcium, PTH, calcitonin, 25-OH-vitD3 and 25,25-(OH)2 vitD3 were normal. He was suspected acquired hypophosphatemic osteomalacia, and treated with oral phosphorus (3 g/day) and calcitriol (0.25 mg/day). Firstly, we had performed PET-CT scan of whole body, but did not detect any sign of tumor. Therefore, he was taken MRI of whole body, and detected mass lesion in only his right wrist (Figure 2). Gadolinium enhanced MRI image showed non homogeneous high intensity lesion was detected along radius, and the mass lesion was enhanced by gadolinium (Figure 2). Biopsy of wrist tumor was performed, and the diagnosis of histology was phospaturic mesenchymal tumor. Histological finding revealed that the tumor was composed of small, spindle to oval cells. There were not clusters or multinuclear cells. The tumor had microcystic areas and poorly formed cartilaginous foci. The margins of the tumor appeared well delimited from the surrounding fibrous tissues. Thereafter, the wrist tumor was resected. Two weeks after removing the tumor, the biochemical parameters including serum phosphate and alkaline phosphatase were improved. The serum concentration of FGF 23 was also normal (45.1 pg/mL).

 After 8 months following tumor resection, revision THA was performed (Figure 1(d)). Walking with full weight bearing was allowed immediately. Finally, He was able to obtain free gait without pain.

## 3. Discussion

Osteomalacia is a generalized mineralization disorder of the osteoid matrix, consisting of a deficit in calcium and phosphate incorporation [4, 5]. Accumulation of non-mineralized osteoid induced bone fragility and caused clinically as pain and bone deformity, muscle weakness, and hypocalcemia. In the growing skeleton, the metaphyses are also affected, causing abnormal growth [3]. 

FGF23 reduces serum phosphate by inhibiting proximal tubular phosphate reabsorption through decreased expression of type 2a and 2c sodium-phosphate cotransporters [6]. At the same time, FGF23 reduces serum 1,25(OH)2D by inhibiting the expression of 25-hydroxyvitamin D-1ahydroxylase and also stimulating the expression of 25-hydroxyvitamin D-24-hydroxylase [6]. Because 1,25(OH)2D enhances intestinal phosphate absorption, FGF23 inhibits intestinal phosphate absorption through its effect on vitamin D metabolism [6]. Therefore, FGF23 is a physiological regulator of phosphate, and vitamin D metabolism.

 In some studies [7, 8], FGF23 levels were determined by immunoassay before and after tumor resection. These reports concluded that FGF23 is increased in many patients of osteomalacia prior to tumor resection. However, the tumor was resected, the renal function and serum FGF23 levels usually return to normal within 24 h [9]. Therefore, quantification of serum FGF23 level is critical to settle the diagnosis and the existence or absence of residual tumor [8]. 

**Figure 3:**
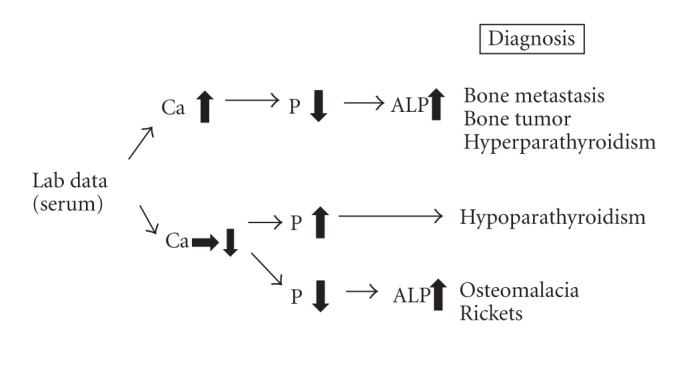
Scheme of differential diagnosis of bone metabolic disease.

In our case, the diagnosis was settled by histological finding. Actually, the serum levels of 25-(OH)2 vitD3, FGF23, phosphate and alkaline phosphatase were improved after tumor resection. These results are in line with other reports. The prosthesis migration should be caused by osteomalacia due to FGF23-induced tumor. Typical radiological features of oncogenic osteomalacia are Looser-Milkman fracture (multiple, spontaneous, and idiopathic fractures). However, the fracture was caused by obvious injury, and femoral bone density at the time of fracture was normal in this case. Further, intraoperative findings did not show the evidence of osteomalacia. The only key information to figure out the diagnosis of this case was the data of blood examination prior to first operation, low serum phosphate, and increased alkaline phosphatase. We provided the screening protocol of differential diagnosis for bone metabolic syndrome by lab data in Figure 3. We have to consider blood examination including phosphorus metabolism. In conclusion, we experienced a rare case of acute prosthesis migration after femoral replacement due to osteomalacia by FGF23-induced tumor.
